# Molecular epidemiology of hepatitis B virus among HIV co-infected and mono-infected cohorts in Northwest Ethiopia

**DOI:** 10.1186/s12985-022-01774-6

**Published:** 2022-03-24

**Authors:** Yeshambel Belyhun, Uwe Gerd Liebert, Melanie Maier

**Affiliations:** 1 Department of Virology, Institute of Medical Microbiology, Leipzig University, Leipzig, Germany; 2 School of Biomedical and Laboratory Sciences, College of Medicine and Health Sciences, University of Gondar, Gondar, Ethiopia

**Keywords:** HBV, Genetic diversity, HIV/HBV co-infection, Ethiopia

## Abstract

**Background:**

Hepatitis B virus (HBV) infection is a particular concern in human immunodeficiency virus (HIV) infected individuals. In Ethiopia, detailed clinical and virological descriptions of HBV prevailing during HIV co-infection and symptomatic liver disease patients are lacking. The aim of this study was to investigate HBV virological characteristics from Ethiopian HBV/HIV co-infected and HBV mono-infected individuals.

**Methods:**

A total of 4105 sera from HIV positive individuals, liver disease patients, and blood donors were screened serologically for HBV. The overlapping polymerase/surface genome region of HBV from 180 infected individuals was extracted, amplified, and sequenced for genotypic analysis.

**Results:**

The HBsAg seroprevalence was detected 43% in liver disease patients, 8.4% in blood donors, and 6.7% in HIV/HBV co-infected individuals. The occult HBV prevalence was 3.7% in HIV/HBV co-infected individuals and 2.8% in blood donors with an overall prevalence rate of 3.4%. A phylogenetic analysis showed three HBV genotypes; A (61.1%), D (38.3%) and E (0.6%). Genotype A belongs to subtypes A1 (99.1%) and A9 (0.9%), but genotype D showed heterogeneous subtypes; D2 (63.8%) followed by D4 (21.7%), D1 (8.7%), D3 (4.3%), and D10 (1.4%).

**Conclusions:**

The HIV/HBV co-infected individuals and blood donors showed lower HBsAg seroprevalence compared to liver diseases patients. Occult HBV prevalence showed no difference between HIV/HBV co-infected and blood donor groups. This study demonstrated predominance distribution of HBV subtypes A1 and D2 in northwest Ethiopia. The observed virological characteristics could contribute for evidence-based management of viral hepatitis in Ethiopia where antiretroviral therapy guidelines do not cater for viral hepatitis screening during HIV co-infection.

## Background

HBV is one of the leading causes of persistent liver infections and chronic liver diseases. HBV infects an estimated 240 million people globally and shows geographical differences in its clinical consequences, seroepidemiological, and genotype distribution [1, 2]. The global HBV distribution ranges from hyperendemicity (> 8%) in areas of east Asia, Africa and the Amazon basins to intermediate (2–7%) in Europe, North America and Australia [1]. HBV is characterized by a high degree of genetic heterogeneity and classified into ten genotypes (A-J) and a number of subtypes [1]. The diversity of HBV genotypes is also related to different clinical infection patterns, liver disease severity [3], viral persistence, and response to antiviral treatments [4].

In terms of transmission routes and associated risk factors, high degrees of epidemiological similarity are common among HBV and HIV [5]. The HBV/HIV co-infected individuals are more likely to transmit hepatitis viruses, and chronic infection with HBV is a frequent clinical phenomenon [6]. In addition to the epidemiological overlap, virological and immunological interactions of HBV/HIV co-infection have also been responsible for changing the natural clinical history and the management of each virus. The introduction of highly active antiretroviral therapy (ART) has reduced the HIV/acquired immunodeficiency syndrome (AIDS) morbidity and mortality. But still, liver diseases associated with viral hepatitis became the second leading cause of death during HBV/HIV co-infection worldwide [7]. In particular, the genetic variations and clinical complications of HBV are believed to be more influenced by the presence of HIV during co-infections [5, 8], although controversy has continued over the impacts of HBV on the natural history of HIV [6, 9]. Chronic HBV/HIV co-infected patients tend to sustain higher levels of HBV viral load in their serum and affect HBV infection resolving mechanisms. More importantly, patients with HBV/HIV co-infection might be at higher risk of chronic liver disease complications, including cirrhosis and hepatocellular carcinoma [6]. As a result, the impact of viral interaction on the natural history and treatment outcomes have become increasingly important in the management of HBV/HIV co-infected individuals especially in regions where both viruses are endemic.

Moreover, treating HBV is less practiced in most African settings, although HIV is associated with a high HBV prevalence in this region [10]. Similarly, HBV was less reported in Ethiopia among liver disease patients during HIV/HBV co-infection, although considerable numbers of HBV seroepidemiological reports were available from blood donors and the general population [11]. In general, detailed clinical and virological descriptions of HBV prevailing during HIV co-infection and symptomatic liver disease patients are lacking in Ethiopia. In particular, a well-established HBV screening and clinical management is ignored in Ethiopia [12]. As a result, clinical parameters are the only means to assess liver related diseases which have limited predictive values to determine impacts of HIV during HBV co-infection. Therefore, the aim of this study was to investigate HBV serological and virological characteristics among large groups of HIV co-infected and mono-infected individuals from northwest Ethiopia.

## Materials and methods

### Study population

The demographic and clinical data and blood sera were collected in 2013 after ethical clearance was granted by the Institutional Ethical Review Board of University of Gondar (Ref. No: RCS/V/P/05/372/2013) and informed consent were obtained from study participants. A total of 4105 study population was enrolled from three health institutions in northwest Ethiopia; namely, University of Gondar teaching hospital, Gondar Health Center and Debretabor district hospital. The study population was recruited from three study groups; blood donors (n = 1720), liver disease patients (n = 252) and HIV carriers (n = 2133) (Table 1). The blood donors were apparently healthy, but the HIV positive group was recruited in a cohort of known HIV positive individuals who were attending ART clinics for their routine follow-ups. ART related clinical information such as ART status and eligibility, ART regimens, clinical ART toxicity assessment, World Health Organization (WHO) staging and status of opportunistic infections were recorded during sera collection. The group of liver disease patients included cases with one or more clinical manifestations of acute and chronic liver diseases patients such as jaundice, ascites, cirrhosis, hepatomegaly, hepatocellular carcinoma and/or liver complications from other infections as reported before [13].

**Table 1:** Frequency distribution of enrolled study population, HBV prevalence in each step of HBV screening among study groups

HBV test done	Study groups, n (%)		
	HIV positive	Blood donors	Liver disease patients
Enrollment	2133 (52.0)	1720 (41.9)	252 (6.1)
HBsAg positive	143 (6.7)	145 (8.4)	109 (43.3)
HBV DNA positive	71 (55.5)^a^	82 (79.6)^a^	98 (89.9)
Sequenced	62 (87.3)	56 (68.3)	59 (60.2)
Total (genotyped)		177* (70.5)	

^a^HBsAg positive sera (15 from HIV positive and 42 from blood donors) were not considered in the next HBV DNA extraction step due to low sera volume. Therefore, the HBV DNA positivity rates were calculated from 128 and 109 sera of HIV positive individuals and blood donors, respectively. *Overall, 180 HBV genotyped cases reproted in the result section by including 3 cases from OHB infection (Table 3)

### Blood collection and serological testing

About 10 ml of venous blood was collected. The sera were screened for HBsAg using commercially available HBsAg rapid test kits (In Tec Products, INC, 332 Xinguang Road, Xiamen, China) according to manufacturers’ instruction. From those HBsAg positive sera, the HBV e antigen (HBeAg) test was done using commercially available enzyme immunoassay kits (Abbott Diagnostics, Wiesbaden, Germany).

For occult HBV (OHB) analysis, anti-HBc antibody (anti-HBcAb) test was done using Enzygnost®Anti-HBc monoclonal ELISA (Siemens Healthcare Diagnostics Products GmbH, Marburg, Germany) among 476 HBsAg negative cases. And then, the anti-HBc antibody positive sera were further tested for anti-HBs antibody using the ARCHITECT system (Abbott Diagnostics, Delkenheim, Germany). Then, the anti-HBs antibody positive and negative sera (but positive for anti-HBc antibody) were used for HBV DNA detection test and viral load determination.

The HIV-1/2 screening was done using the HIV-1/2 rapid test kits according to the Ethiopian national test algorithm adopted from WHO designed for developing countries [14]. The test algorithm used three rapid diagnostic test kits in series; HIV-(1/ + 2) Antibody Colloidal Gold (KHB, Shanghai Kehua Bio-engineering Co Ltd, China) as a screening test, followed by HIV-1/2 STAT-PAK® (Chembio HIV-1/2, Medford, New York, USA) in case of a positive screening result. In case of discordant test results with the above two kits, Unigold™ HIV (Trinity Biotech, Ireland) was used as a tiebreaker to decide the final result.

### DNA extraction, PCR and sequencing

HBV DNA was extracted from 900 µl blood plasma separately using mSample preparation system nucleic acid extraction kits on the Abbott m2000sp automated sample preparation system (Abbott Molecular, Des Plaines, IL, USA). The HBV viral loads were determined using Quantitative Real Time HBV assay (lower detection rate < 10 IU/mL) on the Abbott m2000rt system (Abbott Molecular, Des Plaines, IL, USA). The HBV extraction, amplification and sequencing steps were described in detail before [15].

Briefly, for sequencing, the overlapping HBV polymerase/surface genome region (codons 52–298 nt) was amplified using Taq DNA polymerase (Promega, Madison, WI, USA) using the forward (5′AAAT TCGC AGTC CCA ACC3′) and reverse (5′GCAG CAAA GCCC AAAAG ACC3′) primers as described before [15, 16]. The amplification product was separated using 1.5% agarose gel electrophoresis, excised and purified using Wizard® SV Gel & PCR Clean-Up-System (Promega, Mannheim, Germany). Clean HBV DNA PCR products were subjected to direct sequencing of both forward and reverse strands using BigDye Terminator Cycle Sequencing Ready Reaction kit on the ABI Prism 3500 Genetic Analyzer (Applied Biosystems, Foster City, CA, USA).

### Phylogenetic analysis

After the nucleotide sequences were manually edited and assembled using the Geneious® software version 6.1.4 (http://www.geneious.com), genotyping analysis was done using MEGA 6 (http://www.megasoftware.net). For phylogenetic analysis, HBV genotypes reference sequences were retrieved from the HBV database (https://hbvdb.ibcp.fr/HBVdb/HBVdbNomenclature?nomenclature=table). In addition, a set of homologous sequences was retrieved from GenBank Basic Local Alignment Search Tool (BLAST). The phylogenetic tree was constructed using the Neighbor-Joining method, and the genetic distances were computed using the Kimura 2-parameter method (http://www.megasoftware.net). To confirm the reliability of the phylogenetic tree, a bootstrap resampling test was carried out 1000 times. The HBV nucleotide sequences used in this study were registered to the GenBank/EMBL/DDBJ databases under the accession numbers KT367571-KT367731 and OL630698-OL630716.

### Statistical analysis

Mann–Whitney non-parametric and Chi-square tests were used when appropriate during analysis. Graph Pad Prism version 5.01, 2007 was used for statistical analysis. A P value < 0.05 was considered to be statistically significant.

## Results

### Serological and viral load characteristics of HBsAg positives

**Fig. 1:**
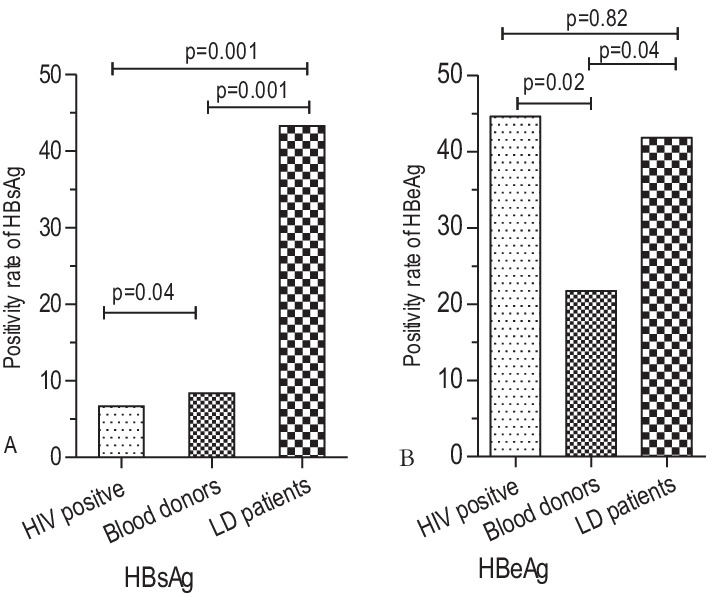
Seroprevalence comparisons of HBsAg (**A**) and HBeAg (**B**) among study groups

Details of demographic and clinical characteristics of HBsAg-HIV co-infected study participants are presented in Table 2. The seroprevalence of HBsAg among HIV co-infected individuals; 6.7% (143) was significantly lower than in blood donors; 8.4% (145) (*p* = 0.04) and liver disease patients; 43.3% (109) (*p* < 0.001) (Fig. 1A). In contrast, the HBeAg frequency was significantly higher among HIV positives (44.6% (25), *p* = 0.02) and liver disease patients (41.8% (23), *p* = 0.04) than blood donors; 21.7% (10) (Fig. 1B).

**Table 2:** Demographic and clinical characteristics of HBsAg seropositive among HIV co-infected patients from northwest Ethiopia

Variables	Seropositive for HBV		Total	X^2^	*P* value
	Non-viraemic	Viraemic			
Age group (in years)					
18–40	42 (47.2)	47 (52.8)	88 (62.2)		
41–61	30 (55.6)	24 (44.4)	54 (37.8)	0.9	0.33
Sex					
Male	30 (46.9)	34 (53.3)	64 (44.8)		
Female	42 (53.2)	37 (46.8)	79 (52.2)	0.56	0.45
WHO staging					
I	41 (47.7)	45 (52.3)	86 (60.1)		
II	5 (29.4)	12 (70.6)	17 (11.9)		
III	6 (60.0)	4 (40.0)	10 (7.0)		
IV	2 (28.6)	5 (71.4)	7 (4.9)	12.1	0.02
Status unknown	18 (78.3)	5 (21.7)	23 (16.1)		
ART status at the time of blood collection					
Naïve	17 (34.0)	33 (66.0)	50 (35.0)		
Experienced	55 (59.1)	38 (40.9)	93 (65.0)	8.2	0.004
Basis for ART eligibility					
Clinical only	14 (70.0)	6 (30.0)	20 (14.0)		
CD4^+^	21 (56.8)	16 (43.2)	37 (25.9)		
CD4^+^ + Clinical	20 (55.6)	16 (44.4)	36 (25.9)	1.3	0.53
ART regimen at the time of blood collection					
d4t-3TC-NVP/EFV	1 (100.0)	0 (0.0)	1 (1.1)		
AZT-3TC-NVP/EFV	29 (52.7)	26 (47.3)	55 (63.2)		
TDF-3TC-NVP/EFV	20 (66.7)	10 (33.3)	30 (34.5)		
3TC/AZT/Kaletra	1 (100.0)	0 (0.0)	1 (1.1)	4.2	0.52
Duration (Months) on ART					
Not started	18 (41.5)	23 (58.5)	41 (28.7)		
1–36 months	8 (25.0)	24 (75.0)	32 (22.4)		
37–73 months	28 (68.3)	13 (31.7)	41 (28.7)		
74–109 months	17 (63.0)	10 (37.0)	27 (18.9)		
> 109 months	1 (50.0)	1 (50.0)	2 (1.4)	16.0	0.007
ART regimens switch from baseline					
No	29 (54.7)	24 (45.3)	53 (57.0)		
Yes	26 (65.0)	14 (35.0)	40 (44.1)	0.98	0.32
Reason for ART regimen change					
Drug toxicity	12 (63.2)	7 (36.8)	19 (46.3)		
Tuberculosis infection	2 (100.0)	0 (0.0)	2 (4.9)		
Clinical/immunological/virological failure	0 (0.0)	1 (100.0)	1 (2.4)		
Other reasons (risk of pregnancy, etc.)	12 (63.2)	7 (36.8)	19 (46.3)	2.9	0.41
ART toxicity encountered					
No	43 (58.1)	31 (41.9)	74 (79.6)		
Yes	12 (63.2)	9 (36.8)	19 (20.4)	0.2	0.69
Immune reconstituted syndrome (IRS)					
No	46 (56.8)	35 (43.2)	81 (56.6)		
Yes	4 (66.7)	2 (33.3)	6 (4.2)		
Not determined	22 (39.3)	34 (60.7)	56 (39.2)	4.7	0.09
Current opportunistic infections					
No	69 (50.4)	68 (49.6)	137 (95.8)		
Yes	3 (50.0)	3 (50.0)	6 (4.2)	0.0	0.97
Previous opportunistic infections					
No	49 (51.6)	46 (48.4)	95 (66.4)		
Yes	23 (47.9)	25 (52.1)	48 (33.6)	0.17	0.68
Tuberculosis treated before					
No	60 (51.7)	48 (50.3)	116 (81.1)		
Yes	12 (44.4)	15 (55.6)	27 (18.9)	0.46	0.50

**Fig. 2:**
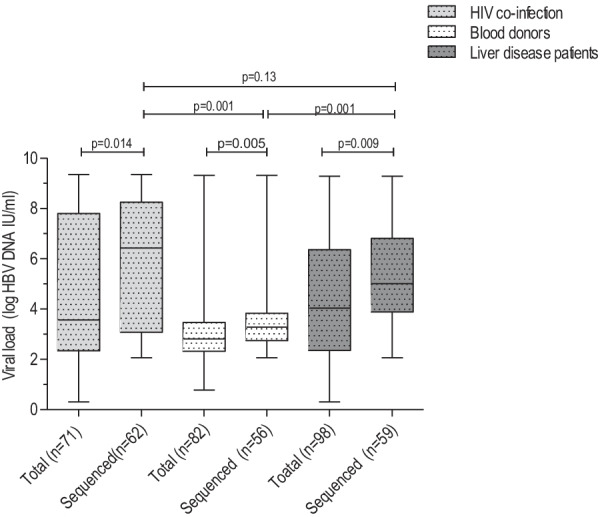
The median HBV viral load levels comparisons among study groups

The HBV viremia rate showed significant differences with a distribution frequency of 55.5% (71/128) in HIV/HBV co-infected individuals, 79.6% (82/103) in blood donors and 89.9% (98/109) in liver disease patients (Table 1). Among HIV/HBV co-infection, HBV viraemic rate was significantly lower among ART experienced; 47.5% (38/80) than ART naives; 68.8% (33/48) (*p* = 0.02). The overall median (Interquartile range; IQR) viral load levels were also compared in each study group and showed significant highest difference in the HIV/HBV co-infected individuals than blood donors and liver disease patients (Fig. 2). Similarly, among the HBV sequenced sera, HIV/HBV co-infected individuals; 6.4 log IU/ml (3.1–8.3) showed higher viral load levels than blood donors; 3.3 log IU/ml (2.7–3.8) and liver disease patients; 5.0 log IU/ml (3.9–6.8) (Fig. 2). However, the median (IQR) HBV viral load levels showed no significant differences between ART experienced; 4.6 log IU/ml (3.1–8.4) and ART navies; 6.5 log IU/ml (2.9–7.9) (*p* = 0.89).

### Serological and viral load characteristics of occult HBV

**Fig. 3:**
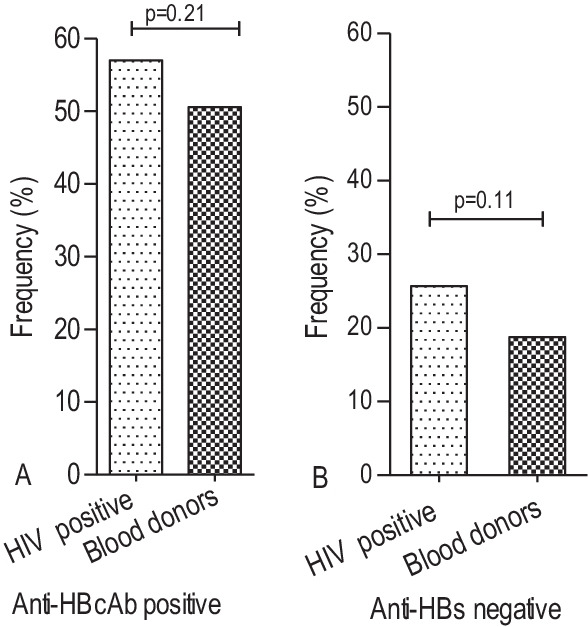
The seroprevalence comparisons of anti-HBcAb positive (**A**) and anti-HBs negative (**B**) markers among HBsAg negative blood donors and HIV co-infected groups. The anti-HBc antibody and anti-HBs tests were not considered among the HBsAg negative sera of liver disease patient due to a relative low study population representation than blood donors and HIV co-infected groups

The anti-HBcAb positivity was 57.0% (171/300) and 50.6% (89/176), respectively among the HBV/HIV co-infected individuals and blood donors, which revealed no statistical significance (*p* = 0.21) (Fig. 3A). The anti-HBs negative frequency among the anti-HBcAb positives showed no significant difference between HIV co-infected individuals; 25.7% (77) and blood donors; 18.8% (33) (*p* = 0.11) (Fig. 3B). Among the anti-HBc antibody positive cases, 14.3% (11/77) from HIV co-infected and 15.2% (5/33) from blood donors showed HBV DNA positivity (Table 3). The overall seroprevalence of OHB infection (HBsAg negative but DNA positive) was 3.4%. However, the seroprevalence became 3.7% (11/300) and 2.8% (5/176) in the HIV co-infected individuals and blood donors, respectively. The viral load for the OHB infection ranges from < 1 to 3.69 log IU/ml.

**Table 3:** Frequency distribution of HBV markers for occult hepatitis HBV among HIV-co-infected and blood donors study groups

HBV markers tested	Study groups, n (%)		
	HIV positive (n = 1990)	Blood donors (n = 1575)	Total (n = 3565)
HBsAg negative enrolled for the test	300/1990 (15.1)	176/1575 (11.2)	476/3565 (13.4)
anti-HBc antibody positive	171/300 (57.0)	89/176) (50.6)	260/476 (54.6)
HBV DNA positive	11/77 (14.3)	5/33 (15.2)	16/110 (14.5)
Total (genotyped)	3/11 (27.3)	0/5 (0.0)	3/16 (18.8)

### Genetic diversity of HBV

An overall of 180 HBV DNA positive sera (177 from HBsAg positive cases and 3 from OHB infection) was sequenced and genotyped (Tables 1 and 3) with a representation of 36.1% (65) HBV/HIV co-infected, 31.6% (56) blood donors, and 33.3% (59) liver disease patients. The genotypic analysis revealed three HBV genotypes; 61.1% (110) genotype A, 38.3% (69) genotype D and 0.6% (1) genotype E. Further sub-genotypic analysis showed genotype A sub-typed as A1, 99.1% (109) and A9, 0.9% (1). Among genotype D, the subtypes distribution showed 63.8% (44) subtype D2, followed by 21.7% (15) subtype D4, 8.7% (6) subtype D1, 4.3% (3) subtype D3, and 1.4% (1) subtype D10.

Genotype A showed no significant (*p* > 0.05) difference distribution among HIV co-infected; 33.6% (n = 37), blood donors; 33.6% (n = 37), and liver disease patients; 32.7% (n = 36). Similarly, genotype D was also distributed with 39.1% (n = 27), 27.5% (n = 19) and 33.3% (n = 23) among the above respective groups (*p* > 0.05).

**Fig. 4:**
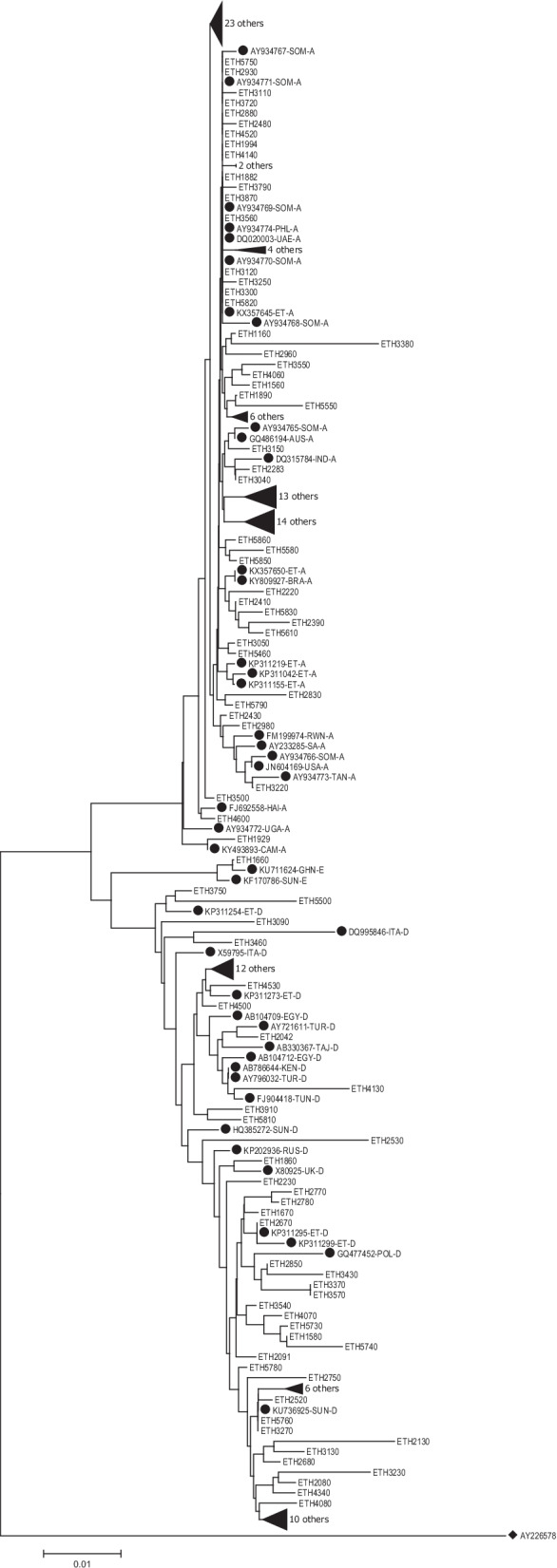
Phylogenetic tree analysis of the HBV sequences isolated among different population category from Ethiopia. The phylogenetic tree was constructed based on the HBV pol/S genome region (codons 52-298). The HBV isolates (denoted by ETH initials) were analyzed with respect to reference sequences retrieved from the GenBank, which are designated by their respective accession numbers (marked as ●) along HBV genotypes and country of origins [AUS-Australia, EGY-Egypt, ET-Ethiopia, GHN-Ghana HAI-Haiti, IND-India, ITA-Italy, KEN-Kenya, OMN-Oman, POL-Poland, RUS-Russia, RWN-Rwanda, SA-S. Africa, SOM-Somalia, SUN-Sudan, TAJ-Tajikistan, TAN-Tanzania, TUN-Tunisia, TUR-Turkey, UAE-United Arab Emirates, UGA-Uganda, UK-United Kingdom]. The genome sequence of the Woolly monkey hepatitis B virus (GenBank AY226578; marked as ♦) was utilized as an out-group for the rooted tree analysis

The phylogenetic analysis revealed that the majority of genotype A clustered within the African subtype, but some were clustered to the Asian, Americas (Haiti) and Australia subtype. Genotype D was commonly clustered to East African, North African, Middle East and European strains. The single isolate genotype E showed the highest homology with African strains (Fig. 4).

## Discussion

According to HBV epidemiological geographical variations [17], Ethiopia can be categorized under the high intermediate (5–7%) to hyperendemicity (≥ 8%) HBV prevalence. The HBsAg prevalence from 6.7% in HIV/HBV infected individuals and 8.4% in blood donors to 43.3% in liver disease patients was recorded in this study. The HBV prevalence from blood donors was in accordance with reports from other African countries [18–20]. Similarly, HBV prevalence in the liver disease patients was comparable to similar reports from symptomatic patients in Africa [21]. The relative lower prevalence of HBV in HIV infected individuals to blood donors and liver disease patients in this study and to other similar studies in Africa (9.0–16.9%) [10, 22, 23] might be partly associated by ART drugs like lamivudine and tenofovir used for HIV therapy during co-infection [24] since the majority of (70.4%) HIV co-infected cases screened for HBV in this study were drug experienced under different ART regimens. Nevertheless, the 6.7% HBV in the current study was within the range of previously reported HBV prevalence (3.7–7.4%) among HIV co-infected individuals in Ethiopia [11]. Moreover, the HBV/HIV co-infected population in the current study was also characterized by a presence of classical and putative immune escape HBsAg gene variants [16] that down-regulate HBsAg-antibody binding surface proteins which usually associated to a high chance of HBV false negativity due low HBsAg [25]. But still, within HBV tested positive cases, the HBV viraemic rate is still high among HIV patients despite ART therapy. This could be associated to the HIV therapy practiced for many years in the study area without testing patients for their HBV status which was responsible for high rate of a combination of multi-drug resistance gene variability observed and reported before [16].

In contrast to lower HBsAg detection rate, however, an almost two times higher HBeAg frequency coupled with significantly higher median viral load levels were observed in these HBV/HIV-infected individuals. Nevertheless, the observation of the significantly higher rate of anti-HBcAb in the HBsAg negative HIV infected study group showed a relatively higher rate of past infection during HIV co-infection. However, the anti-HBs status that showed immunity following past infection were nearly similar from HIV infected and mono-infected groups. This could support the fact that the chance of HBV exposure might not be different among the study groups in the study setting. In Ethiopia, HBV vaccination was introduced in 2007 administered for new born and the current adult study population had no vaccine exposure. Reports also linked to HIV infection to ‘sero-silent’ OHB infections, which presents serious problems for diagnosis, prevention, and control [22]. In this HIV/HBV co-infected individuals of the current study, 3.7% of OHB infection was detected which highlights the need for integrating OHB screening for proper management of liver diseases in HIV co-infected patients.

In this study, the phylogenetic analysis revealed circulation of three genotypes; genotype A, genotype D and genotype E. Overall, genotype A was predominant and both genotypes A and D were the most divergent as they clustered to clades of African, Asian, Americas, Europe, Middle East and Oceania clades. In East Africa, where Ethiopia belongs to, the predominance of genotype A was found in Somalia to the east [26] and in Kenya [27] to the south. However, in neighboring Sudan to the west of Ethiopia, where geographical proximity is close and large influx of population movement through trade and migration are common to the current study settings in northwest Ethiopia, the identified genotypes showed a different distribution pattern. Unlike in Ethiopia where genotype A was predominant, in Sudan genotypes D and E were predominant [19]. Only one isolate with genotype E was identified in this study, but of course with the highest homology to the Sudanese strains.

Although genotype A is known as one of the heterogeneous HBV genotypes [28], a predominance of subtype A1 was identified in this study. This was actually in accordance with most African-derived isolates sequenced so far belonging to subtype A1. The predominance of subtype A1 and the phylogenetic similarity of the majority of the isolates with the neighboring Somalian and other East African isolates also support the notation that this was evolved within the indigenous population of some African countries and had a longer natural history in Africa than other subtypes [29].

Unlike genotype A, subtypes of genotype D were heterogeneous (D1, D2, D3, D4 and D10) in this study population. In fact, subtypes D1 to D5 are known for their wider global distribution among so far described nine subtypes (D1-D9) [1, 28, 30]. In this study, subtype D2 (61.1%) followed by D4 (21.7%) were dominant, although a recent report from blood donors in Ethiopia reported D4 as the least (2.5%) subtype and also failed to identify the subtype D3 [31]. Nevertheless, the subtype D3 was exclusively found among liver disease patients in this study. Previous studies showed subtype D1 was predominant in the Eastern part of African countries [32], despite the current study and an earlier report from neighboring Somalia [29] reported D2 and D4 were predominant. As a result, these subtypes might be speculated to be indigenous in Ethiopia and Somalia. This is because the nearby Middle East and northern African regions [1] were known for the subtype D1, but the frequency of this subtype was very low (8.7%) in the current study. Moreover, the geographic proximity of countries, mostly associated to subtypes D2, D3, and D4 (South Africa, Alaska, south to north-eastern Europe and Oceania) [1, 33] are far distant from east Africa, although few isolates in the current study had a phylogenetic relationship to distant isolates such as from Oman, UK and Italy. Actually, given to the polymerase/surface gene sequences used in the phylogenetic analysis in this study, such conclusion might be partial unless full genome sequences are compared since HIV co-infection is known to be responsible for HBV variability and high rate of recombination [34]. For instance, a recently described new D8 and D9 subtypes from Niger and India, respectively recognized as recombinant forms of genotype D with genotype E [35] and genotype C [36]. A novel hepatitis B virus subtype D10 was also reported circulating in Ethiopia [37], but only a single isolate was identified with this subtype in the current study. Overall, this study showed subtypes of genotype D were heterogeneous in northwest Ethiopia.

## Conclusions

In summary, relative to the blood donors, HIV/HBV co-infected group was characterized by lower HBV seroprevalence, but showed higher HBeAg detection rate and median viral load levels. Moreover, the anti-HBcAb, which showed past HBV infection was significantly higher in HIV co-infected group but showed no difference to anti-HBs (a HBV marker for immunity to past infection) as well as OHB infection. The phylogenetic analysis showed circulation of heterogeneous HBV subtypes (A1, A9, D1, D2, D3, D4, D10 and E) with the predominance of subtypes A1 and D2 in Ethiopia. The HBV subtypes showed the most divergent isolates clustered to both African and non-African clades. This is the first HBV genetic diversity study among HIV co-infection and symptomatic liver disease patients in Ethiopia. These data will have significant public health implications to understand further clinical and virological interplay of HBV during HIV co-infections in this country where viral hepatitis management was totally ignored and HIV antiretroviral guidelines do not even cater for viral hepatitis during HIV co-infection.

## Data Availability

The HBV nucleotide sequences used in this study were registered to the GenBank/EMBL/DDBJ databases under the accession numbers KT367571-KT367731 and OL630698-OL630716.

## Abbreviations

AIDS: Acquired immunodeficiency syndrome
Anti-HBs: Anti- Hepatitis B surface
ART: Active antiretroviral therapy
BLAST: Basic Local Alignment Search Tool
ELISA: Enzyme-linked immunosorbent assay
HBcAb: Hepatitis B core antibody
HBsAg: Hepatitis B surface antigen
HBV: Hepatitis B virus
HIV: Human immunodeficiency virus
MEGA: Molecular Evolutionary Genetics Analysis
PCR: Polymerase chain reaction
OHB: Occult Hepatitis B virus
WHO: World Health Organization
